# Lessons learned from the MOMENT study on how to recruit and retain a target population online, across borders, and with automated remote data collection

**DOI:** 10.1371/journal.pone.0307440

**Published:** 2024-09-16

**Authors:** Andrew Belfiglio, Shayleigh Dickson Page, Sara Pettersson, Michelle van Rijn, Ercole Vellone, Heleen Westland, Kenneth E. Freedland, Christopher Lee, Anna Strömberg, Douglas Wiebe, Subhash Aryal, Barbara Riegel, Tiny Jaarsma

**Affiliations:** 1 University of Pennsylvania Perelman School of Medicine, Philadelphia, PA, United States of America; 2 University of Pennsylvania School of Nursing, Philadelphia, PA, United States of America; 3 Linköping University, Linköping, Sweden; 4 University Medical Center Utrecht, Utrecht, The Netherlands; 5 Tor Vergata University of Rome, Rome, Italy; 6 Wroclaw Medical University, Wrocław, Poland; 7 Washington University School of Medicine in St. Louis, St. Louis, MO, United States of America; 8 Boston College Connell School of Nursing, Boston, MA, United States of America; 9 University of Michigan School of Public Health, Ann Arbor, MI, United States of America; University of Konstanz: Universitat Konstanz, GERMANY

## Abstract

Increasingly, studies use social media to recruit, enroll, and collect data from participants. This introduces a threat to data integrity: efforts to produce fraudulent data to receive participant compensation, e.g., gift cards. MOMENT is an online symptom-monitoring and self-care study that implemented safeguards to protect data integrity. Facebook, Twitter, and patient organizations were used to recruit participants with chronic health conditions in four countries (USA, Italy, The Netherlands, Sweden). Links to the REDCap baseline survey were posted to social media accounts. The initial study launch, where participants completed the baseline survey and were automatically re-directed to the LifeData ecological momentary assessment app, was overwhelmed with fraudulent responses. In response, safeguards (e.g., reCAPTCHA, attention checks) were implemented and baseline data was manually inspected prior to LifeData enrollment. The initial launch resulted in 411 responses in 48 hours, 265 of which (64.5%) successfully registered for the LifeData app and were considered enrolled. Ninety-nine percent of these were determined to be fraudulent. Following implementation of safeguards, the re-launch yielded 147 completed baselines in 3.5 months. Eighteen cases (12.2%) were found fraudulent and not invited to enroll. Most fraudulent cases in the re-launch (15 of 18) were identified by a single attention check question. In total, 96.1% of fraudulent responses were to the USA-based survey. Data integrity safeguards are necessary for research studies that recruit online and should be reported in manuscripts. Three safeguard strategies were effective in preventing and removing most of the fraudulent data in the MOMENT study. Additional strategies were also used and may be necessary in other contexts.

## Introduction

The internet has increased the ability to reach patients who may otherwise be missed in traditional point-of-care clinic settings and thereby has demonstrated its potential as a tool for improving public health and medical care. It is also a powerful tool for the conduct of health care research. Increasingly, researchers are turning to online survey applications and platforms like social media to recruit, enroll, and collect data from participants [1–5].

Virtual or online research methods have appeal for both researchers and participants in that they may remove barriers to engaging in research, improve convenience of data collection, and increase resource efficiency [5]. In-person healthcare touchpoints are the dominant and conventional opportunities to identify potential study participants and complete research assessments. For example, persons with chronic heart failure may be recruited to a research study while in the waiting room for their cardiology appointment. Through ease of access, greater convenience, and less reliance on institutional pathways, conducting research online may improve the inclusion of patients who live in areas with a shortage of providers, or who face other barriers to accessing the healthcare system such as difficulty with transportation, mobility, social support systems, or uninsurance or underinsurance [3, 6, 7].

Online research studies can improve convenience for patients and research teams by allowing asynchronous activities [8–10]. Patients do not have to align their schedules with researchers in order to enroll and participate in studies. For minimal risk studies, it is possible for participants to “self-enroll” and respond to assessments on their own time and from any location. This not only improves convenience for participants but also saves valuable research resources such as staff time, data collection materials, office/lab space, and remuneration for participant transportation and parking. Further, online research can be designed to efficiently enroll participants and collect data from national and international populations.

With the potential to reach billions of individuals across the globe, researchers are increasingly using social media (e.g., Facebook, Twitter) for participant recruitment [2–4]. Facebook is now the largest web-based social media platform, with about 2.6 billion users worldwide [11]. In the USA alone, there are approximately 223 million Facebook users, most of whom access the site at least once a day, and Twitter (recently renamed as X) has over 550 million users worldwide [12]. The utility of social media recruitment for a research study depends on the population being recruited and the goals of the study. Topolovec-Vranic and Natarajan found that social media recruitment was the most effective recruitment method for just 40% of studies reviews [4]. Recruitment through social media may improve access to “hard-to-reach” populations (e.g., people who use drugs) or individuals with specific diagnoses [3, 4]. However, those recruited through social media may differ from the general population in key demographic characteristics. Namely, participants recruited through social media tend to be younger compared to traditional recruitment methods [2–4]. The cost of recruitment through social media is highly variable as researchers may purchase paid ads that target specific demographics, use unpaid posts to share recruitment materials, or a combination. While paid advertisements at Facebook for health-related study recruitment have increased [13], the cost-effectiveness of using paid ads versus unpaid posts on Facebook is unclear [2]. Most research reporting on social media recruitment has been conducted in the United States; less is known about the strengths and weaknesses of using social media to recruit international populations.

Through social media advertising and the automation of research activities, researchers may be able reach more participants and increase the efficiency of their research staff time. However, researchers must carefully consider the study design and recruitment strategy to reach the target population and prevent fraudulent data from being submitted. Several authors have published experiences and recommendations for protecting data integrity [1, 14–16]. Teitcher et al., describe strategies to detect and prevent fraudulent data at four levels: the questionnaire or survey instrument (e.g., include duplicate questions and monitor responses for consistency), the participant’s non-questionnaire data (e.g., check for similar email addresses), the computer information (e.g., track IP addresses), and the study design (e.g., reducing emphasis on the compensation) [1]. Further, following suspected fraudulent activity, Pozzar et al used a similar set of data quality indicators and found that 94.5% of submissions were fraudulent [14]. The work to date has shown that fraudulent data can be a significant problem in cross-sectional observational studies.

In conducting the MOMENT (SyMptOMs and SElf-Care iN Chronic Illness ManagemenT) study–a multinational, longitudinal study in which we recruited and enrolled adults with chronic conditions to use a mobile app twice daily–we received an influx of fraudulent data that demonstrated the increasing sophistication of perpetrators. In this paper, we use this experience to (1) describe important methodological considerations regarding the use of apps and automating data collection across different countries, (2) identify challenges to reaching participants through social media internationally, and (3) evaluate the effectiveness of implementing strategies to prevent bots and related threats to data integrity in longitudinal studies.

## Methods

MOMENT used ecological daily assessment (EDA) over a two-week period to measure symptoms of chronic diseases, self-care management behaviors, and the factors that influence decisions about self-care. EDA is a data collection technique in which the participant’s behaviors (i.e., self-care in response to symptoms) are repeatedly sampled in approximately real time [17]. Eligibility criteria of the MOMENT study included having one or more of the following chronic conditions: arthritis, asthma, chronic obstructive pulmonary disease, heart failure, and/or diabetes; having at least 3 months of experience with the condition(s); and experiencing bothersome symptom(s) at least three times weekly. MOMENT was designed as an international study because responses to symptoms have been shown to differ between countries [18]. In this study, participants lived in Italy, the Netherlands, Sweden, or the United States, because the research team members were located in these countries. Data were collected between May 4, 2022 and September 30, 2022.

After completing baseline surveys about health and self-care management behaviors (i.e., actions taken in response to symptoms), participants completed two weeks of EDA. Using an app on the participant’s own smartphone, participants were prompted to complete twice daily (11am & 7pm, local time) reports about their most bothersome symptom for two weeks. These times were considered least burdensome and most likely to receive a response (i.e., least likely times for a participant to be unavailable due to sleep, commuting, or typical meal times). With each prompt, participants reported (i) the characteristics of the symptom (severity, bothersomeness, frequency), (ii) their interpretation of the cause of the symptom, (iii) their response to the symptom (which could include either certain self-care management behavior(s) or no response), and (iv) why they chose that action (or inaction). At the end of the two weeks, participants completed a survey on the acceptability of the mobile app and had the option to complete an additional individual phone interview about their experience.

### Apps and automation

After receiving Institutional Review Board approval from the University of Pennsylvania, the team set up enrollment and data collection procedures that were initially fully automated. The social media recruitment flyers (described below) directed participants to REDCap (Research Electronic Data Capture) to complete eligibility screening, informed consent, and baseline surveys. REDCap is a secure, web-based software platform designed to support data capture for research studies, providing an intuitive interface for validated data capture and automated export procedures for seamless data downloads to common statistical packages [19, 20]. The Multi-Language Management module was used to provide the baseline survey in four different languages simultaneously. This allowed data entered by participants in four different languages to be maintained in a single database. English was designated as the base language and translations in Italian, Dutch, and Swedish were provided by the native speaking study team members. Completion of the baseline survey prompted an automated transition to LifeData experience sampling software, where participants then entered twice daily EDA data for two weeks. Four separate “LifePaks” were created to assess the daily symptom monitoring questions in Italian, Dutch, Swedish, and English through prompts delivered via push notification to participants’ smartphones.

**Fig 1:**
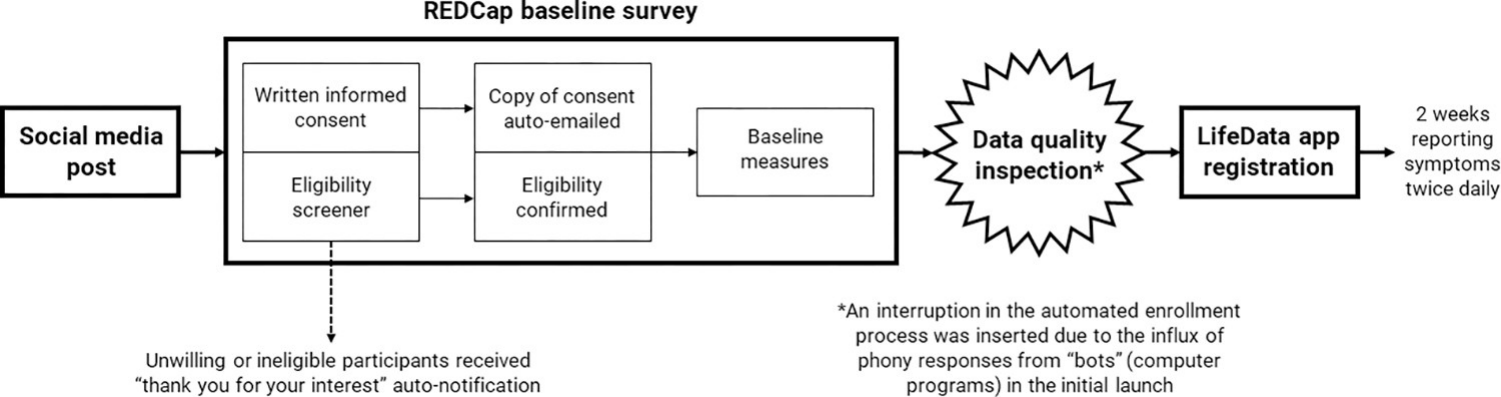
Flowchart depicting the automated process of data collection.

Data collection was automated by a number of processes in REDCap and LifeData (see Fig 1). The rationale for automation was that an immediate transition from the baseline survey to downloading the mobile app would reduce study attrition. After completing the REDCap baseline survey, participants were automatically re-directed to download the LifeData app. The link enabled participants to install the LifeData app from their phone’s app store and, upon successful installation, immediately complete the start-up session where they selected their condition and the symptom they wished to report on for the next two weeks. Participants then received a mobile push notification two times a day for two weeks. Clicking on the push notification or opening the LifeData app triggered a series of multiple choice and free-text questions within the app. Thorough testing was required to ensure all automation and translation functioned appropriately. Translations were important to test because the meaning of words and phrases can change once seen in the context of the survey. This was especially true for user interface language, such as “next,” “required,” and “submit,” as well as the error messages and other system notifications. All interface language was available to translate in the Multi-Language Management module. All members of the research team and some invited colleagues, friends, and family members from each country participated in testing the survey to ensure automation and translation was appropriate and clear.

### Participant recruitment

We aimed to recruit all participants via social media, defined as websites or mobile apps that enable users to create and share information and to build virtual communities [21]. Social media was chosen for its wide reach and demographic diversity, as opposed to crowdsourcing platforms which may not reach as broad of a population. Variations of the advertisement were created with condition-specific content (to share through medical condition-specific organizations) as well as with content that included all of the relevant medical conditions (to share through organizations that include people with diverse conditions). Each advertisement included a QR code or link to REDCap.

Three general approaches to recruitment were designed: (1) flyers distributed through the social medial channels of patient support organizations, (2) flyers posted directly to social media, and (3) paid advertisements. Research staff in each country tailored the recruitment approaches for each local context and flyers were posted in the local language (i.e., Italian, Dutch, Swedish, English).

In all four countries, patient support organizations were identified via internet search and asked to assist in sharing the study flyer through social media channels (e.g., Facebook, Twitter/X, Instagram).

In Sweden and the US, flyers were also posted directly to Facebook. The search function on Facebook was used to identify Groups (i.e., public or private online communities centered around a topic), and Pages (i.e., public profiles of organizations) where adults with one of the five chronic conditions exchange support and information about their chronic condition(s). Depending on the preferences and rules of the Group or Page, the flyer was posted either by the researcher or the administrator of the Group or Page. In addition, study team members posted the study flyer on their personal Facebook and Twitter/X pages; these posts could then be shared or retweeted.

In the US, paid advertisements were posted on Facebook and visible to Facebook users older than 18 years old residing in the US. The paid advertisements were closed after 24 hours due to technical errors with posting that Facebook support was unable to resolve. The ads were said to violate Facebook’s terms, but no other details were given, and attempts to reach Facebook customer support went unanswered.

Recruitment took place between March 2022 and September 2022. Remuneration for participation was provided via an Amazon gift card. A maximum of 30 US dollars could be earned by completing the study, an amount that was chosen by the study team based on past research experience across the four countries and participant feedback from a small pilot study (data not included). Using loss aversion principles from behavioral economics [22], we structured the remuneration plan such that participants started with 30 US dollars in an electronic bank and $1 was deducted for each missed prompt.

### Data integrity safeguards

Within 48 hours of the initial launch of the study, an influx of fraudulent responses was received from suspected “bots”–computer programs created by individuals or groups who try to siphon research dollars by “completing” studies and receiving the participant compensation. In response, the team temporarily closed the surveys, and the safeguards described below were incorporated into the REDCap baseline survey.

**Fig 2:**
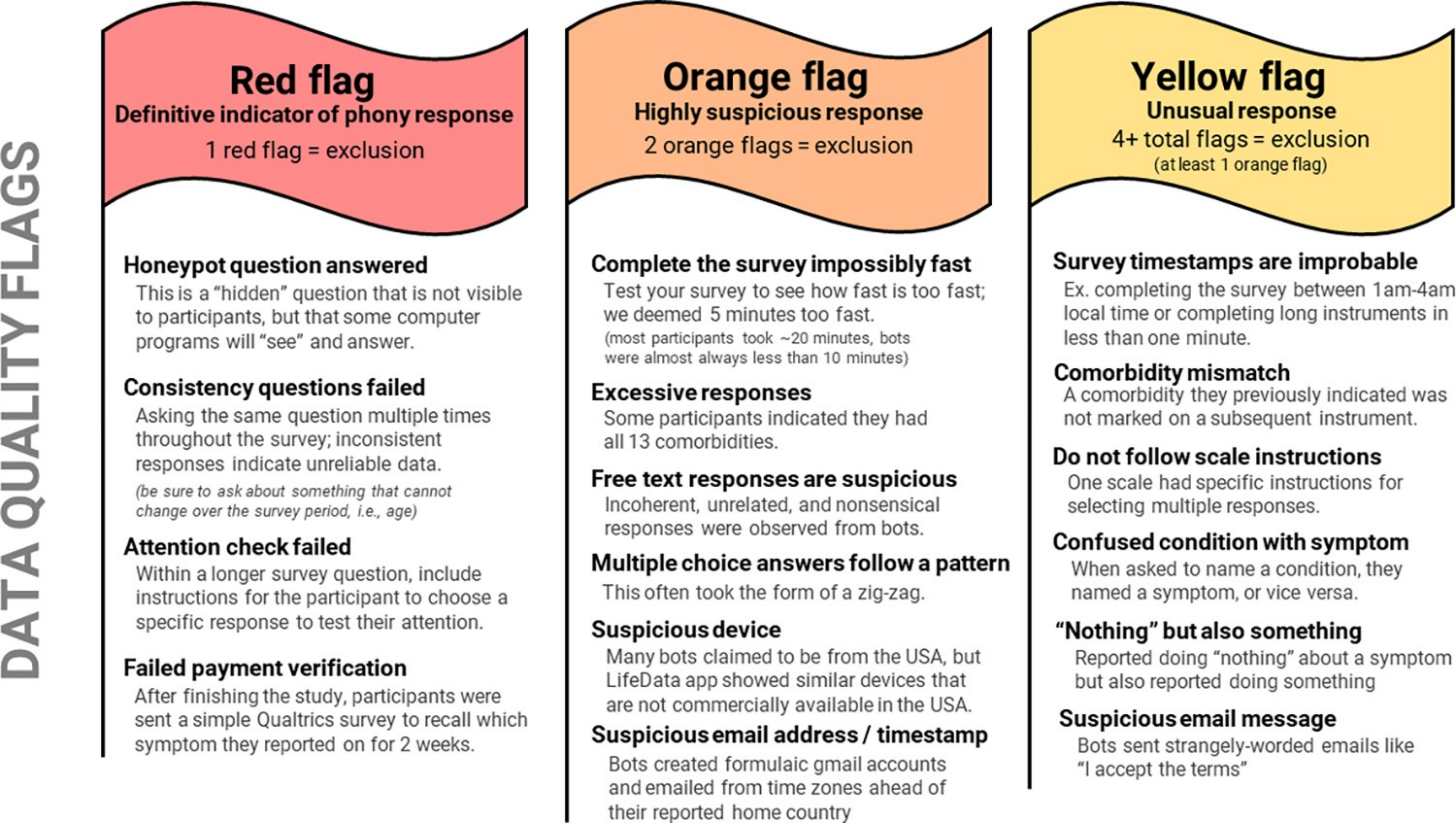
Data quality flags to identify invalid data.

First, the REDCap administrator at the University of Pennsylvania enabled the reCAPTCHA (Completely Automated Public Turing test to tell Computers and Humans Apart) feature, which requires participants to check a box or answer questions to prove their legitimacy before proceeding to the baseline survey. Second, honeypot, attention check, and consistency check questions were added to the REDCap survey to help identify bots and respondents who provided invalid data (see definitions in Fig 2). Third, the automatic redirect from REDCap to LifeData was removed (see Fig 1). Instead, the researchers performed a manual inspection of the REDCap responses using prespecified criteria, described below. Only participants who passed this data quality inspection were invited to complete the longitudinal portion of the study on the LifeData app. Lastly, a payment verification survey, which was created using another data collection software (Qualtrics), was used to add a second CAPTCHA test and to ask the participant to recall which condition and symptom they reported on daily for the past two weeks. The payment verification survey was emailed to participants after completing the study as an additional precaution to avoid including data from and paying participant incentives to bots. This addition was motivated by the observed sophistication of bot responses (i.e., the ability to temporarily retain memory of their previous responses within the baseline survey) in the first launch. Since response initiation times to the EDA app prompts (i.e., the time of push notification to the time of EDA session initiation) in the initial launch did not display a recognizable pattern, a fast response to the push notification was not considered a flag.

The hidden honeypot question asked “In what country do you live?” to appear like a real question. The attention check instructed the participant to “rate this question as ’fair’,” with the instructions nested within a longer question that followed the length and format of others around it. The consistency check asked “What is your age?” multiple times throughout the survey.

The manual inspection criteria (i.e., data quality flags) for REDCap and LifeData responses were created based on patterns of fraudulent responses received in the initial launch, the team’s past data collection experience, and a blog post by a researcher with similar experience [23]. Yellow, orange, and red “flags” were assigned to each criterion based on how certain we were that the response indicated a fraudulent response (see all criteria in Fig 2). Yellow indicated an unusual response, orange indicated a suspicious response, and red was a definitive indicator of a fraudulent response. Participants were considered a ‘reasonable exclusion’ if they had: (a.) any red flags, (b.) two or more orange flags, (c.) four or more flags, one of which was orange, or (d.) failed the payment verification survey. Thresholds for exclusion were intended to be conservative in that it should be more likely to include a convincing fraudulent response than to exclude a legitimate respondent.

### Inclusivity in global research

Additional information regarding the ethical, cultural, and scientific considerations specific to inclusivity in global research is included in the Supporting Information (S1 Checklist).

## Results

### Effectiveness of data integrity safeguards

Within 48 hours of the initial launch, 411 responses to the REDCap baseline survey were received. Over 99% of these responses were later determined to be fraudulent, which prompted the redesign of the data collection methods and relaunch of study recruitment. Impressively, of the 411 responses received, 265 (64.5%) successfully registered for LifeData using their ID number provided by REDCap and entered EDA data over the two-week period, demonstrating the sophistication of the bots to participate in longitudinal research. This may have been accomplished through a phone farm–a network of programmed mobile devices–since each LifeData app installation requires a singular device. Only 7 of 411 (1.7%), however, passed manual inspection criteria, and only 2 (0.5%) passed payment verification. The two respondents who passed inspection and payment verification were considered legitimate and included in the final sample. All fraudulent responses selected the English (US) version of the survey; however, geolocation was not collected so the location of the bots cannot be known.

After adding the reCAPTCHA feature to the REDCap baseline survey, responses decreased from 411 in 48 hours (~8.5 responses per hour) to 147 in 3 months (<0.1 responses per hour). Manual inspection in the re-launch found that 11.6% of records (17 of 147) included invalid data and as a result were not invited to register in LifeData. Fifteen of these cases were identified through a single attention check question wherein the participant was told which answer to select, but they did not select that answer. The remaining two flagged cases were identified through a combination of excessive comorbidity (i.e., every comorbid condition is selected in checklist of 13 conditions), suspicious pattern of responses (i.e., zig-zag pattern through a matrix question set), and suspicious responses to open-ended questions (i.e., non-sensical, unrelated, or unusual language).

### Recruitment and retention

**Fig 3:**
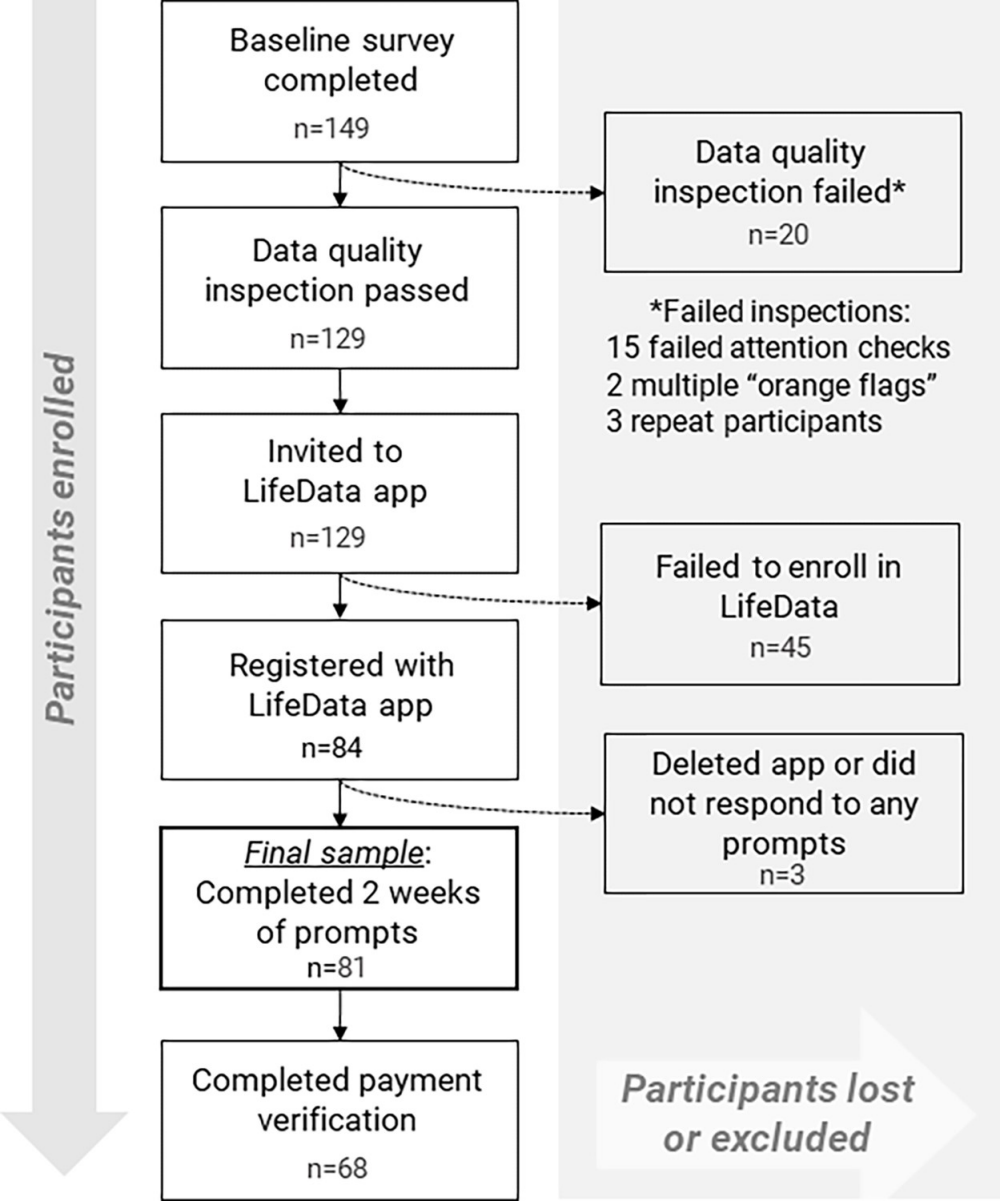
Recruitment & retention after implementation of data quality safeguards.

During the relaunch, a total of 147 responses were received in the REDCap baseline survey. After manual data inspection, 127 (86.4%) were determined to have valid data and were invited to LifeData to report on symptoms for two weeks (see Fig 3). Of those invited to LifeData, 81 (63.8%) successfully completed registration, received twice daily prompts for two weeks, and were considered enrolled in the study. Ninety-six percent of enrolled participants responded to at least one prompt and the majority (81%) responded to at least 75% of the prompts.

Combining valid responses from the initial launch and relaunch phases, all of the participating countries were represented, though participation was lower in the US. Italy (n = 22), the Netherlands (n = 25), Sweden (n = 24) and the United States (n = 10) accounted for the total 81 enrolled participants. Each of the five chronic conditions were represented by participants from at least three of the four involved countries. The recruitment, retention, and exclusion of participants of the combined initial launch and relaunch is summarized in Fig 3. Demographic characteristics of participants in the final sample are provided in Table 1.

**Table 1:** Demographics of final sample (N = 81).

	n (%)
**Age**	
mean(sd)	49 (15)
Range	20–80
**Gender**	
Female	62 (76.5)
Male	18 (22.2)
Prefer Not to Answer	1 (1.3)
**Country**	
The Netherlands	25 (30.9)
Sweden	24 (29.6)
Italy	22 (27.2)
United States of America	10 (12.3)
**Condition** *	
Arthritis	16 (19.8)
Asthma	35 (43.2)
COPD	12 (14.8)
Diabetes Mellitus	26 (32)
Heart Failure	11 (13.6)
**Employment**	
Student or Trainee	3 (3.7)
Full Time	24 (29.6)
Part Time	18 (22.2)
Unemployed	3 (3.7)
Unable to work due to illness/disability	17 (21.0)
Retired	16 (19.8)
**Finances**	
Have enough to make ends meet	60 (74.1)
Do not have enough to make ends meet	6 (7.4)
Have more than enough to make ends meet	15 (18.5)
**Race (n = 10)** †	
White	7 (70)
Black	2 (20)
Native American/Alaska Native	1 (10)
**Ethnicity (n = 10)** †	
Non-Hispanic	10 (100)

* Participants could select more than one condition

† The Italian, Dutch, and Swedish versions of the survey did not ask for race and ethnicity as this is not customary in those countries.

With regards to the acceptance of gift card incentives to participate in research, 16 of 81 participants (19.8%) did not complete the payment verification survey to receive the gift card. During optional post-interviews, some European participants found it strange to be paid for research and others did not like Amazon (the gift card provided), which may explain non-completion. Of the 65 who completed payment verification and to whom gift cards were emailed, only 51 (78.5%) had redeemed the gift card as of six months post-study closure.

### User experience

At the end of the study, some of the participants indicated their willingness to schedule a brief interview to share their experiences with the app and were contacted by the researcher. The participants from Italy and US did not respond when contacted, four Dutch participants and one Swedish participant consented and were interviewed. All interviewees reported that the app was easy to use and the questions were clear. They said that the twice-daily push notifications prompting them to report their symptoms in the app were helpful. Four participants felt that the timing of the push notifications was convenient as they occurred around lunch time and around dinner time. One participant appreciated the two-hour response window as it allowed them to postpone the response to a time that was more convenient for them. All participants reported that the time required to respond was acceptable and took no longer than 5 minutes. Some participants felt forced to choose an answer that did not completely fit their symptoms and response. Almost all participants indicated that they would have preferred open-ended questions in the app. One participant noted the absence of questions about mental state was problematic because the perception of symptoms can be related to this.

## Discussion

This paper describes lessons learned about automating data collection across countries, recruiting participants through social media internationally, and protecting data integrity while conducting a longitudinal observational study on self-care of chronic illness. The need for data integrity safeguards in all online research has been reported in the literature and is further highlighted by the results of the MOMENT study. First, the ubiquity of “bot” computer programs written to take online surveys to collect gift card incentives is demonstrated in the over 400 responses received in the first 48 hours of the initial study launch (8.6 responses per hour). The use of CAPTCHA or reCAPTCHA is essential for preventing bots from entering a research study that permits automated remote enrollment. After the REDCap administrator enabled the CAPTCHA feature, the survey response rate normalized. However, even after implementing the CAPTCHA safeguard, a considerable proportion of responses were still deemed invalid and were excluded from the final sample. Most of the responses with invalid data were identifiable with a single attention check question. However, we also detected invalid data from multiple data quality flags. It is possible for a participant to unknowingly guess the correct answer to an attention check question, so it is critical to include multiple safeguards beyond a single attention check question. The manual data inspection process we used–inspecting the REDCap baseline survey for data quality flags before inviting participants to continue to the LifeData app–was critical for minimizing data integrity threats to this longitudinal study. Inclusion of the post-study payment verification survey provided a final safeguard prior to remuneration and reassurance that the longitudinal data were valid. These processes were essential to protect against bots but can also extend to human respondents who provide fake data.

When publishing the protocol or the results of a research study that recruited participants online and allowed participants to self-enroll, authors should report the multiple specific safeguards implemented to ensure data integrity. The extent of fraudulent responses in this study was similar to that of Pozzar et al. [14] This constitutes a significant threat to data integrity in online research. Given the general lack of safeguards reported in current literature, it is possible that published results contain invalid data. This has likely resulted in both Type I and Type II errors in recent scientific literature. The current investigators were alerted to the data integrity threat by the influx of survey responses that we received, but bots may become more sophisticated to elude detection. Therefore, researchers must be proactive to support data integrity and strategies will have to evolve over time in order to address increasingly sophisticated threats to data security as they emerge.

These data integrity considerations are important because automating study procedures and recruiting participants through social media can be highly efficient. The MOMENT study recruited participants from age 20 to 80, with one of five chronic conditions, across four countries. This shows that recruiting through social media can reach a wide variety of participants. However, in this study, recruitment on social media was more effective in the three European countries compared to the US. One reason for the difference in effectiveness of social media recruitment outside of the U.S. could be the remuneration amount that may have been lower than what participants expected for what was required of them in this study [24].

Participants reported that the study procedures were acceptable and convenient, except that they wanted more open-ended questions for reporting on their symptoms because their situations can be complicated. A few participants had trouble registering for the LifeData app to receive daily smartphone prompts. This was remedied by providing step-by-step instructions with screenshots as a troubleshooting guide. Retention was satisfactory, in that more than half of baseline respondents continued to report twice daily symptom occurrence for two weeks, with high rates of responses to the daily prompts, despite a relatively modest compensation of $30 US dollars. This result could underscore the user-friendly design of the data collection procedures or the increased study buy-in associated with recruiting through trusted patient organizations and online communities. Considering the challenges that researchers have faced in adequately recruiting a sample from social media platforms [25], our strategy in recruiting exclusively from social media can be replicated in future studies while simultaneously strengthening the integrity of data sources derived from online research.

## Conclusion

The lessons learned from the MOMENT study can help others to effectively design online studies and avoid common challenges associated with apps and automating data collection, reaching participants online, and preventing bots and related threats to data integrity. While employing apps and automation offers tremendous benefits, rigorous testing of the instruments and providing troubleshooting guides for participants are essential features for ensuring that participants will be able to conveniently enroll and participate in the research. Additionally, though recruiting online can help more participants find a study, it can also be found by “bots”–thus threatening the validity of the data. Lessons learned for automated, online data collection include: (1) thorough testing and revision to ensure participants will complete study procedures as expected; (2) a comprehensive data integrity plan, including monitoring plans and safeguards integrated into data collection such as CAPTCHAs and questions to flag threats to data validity; and (3) a readiness to troubleshoot, which includes a way for participants or the data collection system to alert the research team if they experience difficulty and, and ensuring that the team is prepared to make adjustments accordingly.

## Supporting information

S1 Checklist Inclusivity in global research questionnaire. (DOCX)
